# Post-exercise differential response of central and brachial blood pressure in patients with coronary artery disease: A randomized crossover trial

**DOI:** 10.1371/journal.pone.0317212

**Published:** 2025-02-21

**Authors:** João Luís Marôco, Vitor Angarten, Rita Pinto, Vanessa Santos, Bo Fernhall, Helena Santa-Clara, Xavier Melo

**Affiliations:** 1 Integrative Human Physiology Laboratory, Manning College of Nursing & Health Sciences, University of Massachusetts Boston, Boston, MA, United States of America; 2 Faculdade de Motricidade Humana–Universidade de Lisboa, CIPER ‐ Centro Interdisciplinar de Estudo da Performance Humana, Lisboa, Portugal; 3 Exercise and Cardiovascular Rehabilitation Laboratory, Centro Cardiovascular da Universidade de Lisboa (CCUL), Faculdade de Medicina, Universidade de Lisboa, Lisboa, Portugal; 4 KinesioLab, Research Unit in Human Movement Analysis, Instituto Piaget, Almada, Portugal; 5 Centro de Investigação Interdisciplinar Egas Moniz (CiiEM), Egas Moniz School of Health & Science, Caparica, Almada, Portugal; University of Perugia, ITALY

## Abstract

The post-exercise hypotension response is controversial among patients with coronary artery disease (CAD). Factors behind this disparity may include post-exercise differential effects on central and brachial blood pressure (BP), exercise intensity and inter-individual variability. Thus, we investigate group and individual central and brachial BP responses 5, 15 and 30-min after combined exercise of different intensities in participants with and without CAD. Seventeen participants with stable CAD and eighteen aged-matched controls (52–81 years) completed an acute bout of high and moderate-intensity combined exercise. Brachial and central systolic (cSBP) pressures were assessed via oscillometry and carotid tonometry, respectively. Central pulse wave velocity was also measured. Group mean changes were examined with linear mixed models, and bSBP and cSBP post-exercise individual responsiveness quantified via the region of practical equivalence and highest density interval, a Bayesian decision rule. Regardless of exercise intensity, cSBP was persistently increased during recovery in participants with CAD (difference 30 –baseline (d_30-bas_) = 10, 95% CI: 4 to 17 mmHg, *p* = 0.001) but reduced in controls (d_30-bas_ = -13, 95% CI: -19 to -7 mmHg, *p* = 0.003). bSBP was unchanged in both groups (CAD: d_30-bas_ = 1, 95% CI: -3 to 6 mmHg, *p* = 0.995, control: d_30-bas_ = -4, 95% CI: -2 to 8 mmHg, *p* = 0.999). Most participants with CAD exhibited sustained elevations in cSBP (n = 10), while most controls were post-exercise hypertensive responders (n = 11) with changes >|5| mmHg. We found differential post-exercise effects on central and brachial BP independent from combined exercise intensity but not clinical population.

**Clinical trials.gov registration ID**: NCT06617117

## Introduction

High blood pressure (BP) is the main risk factor in the pathogenesis of coronary artery disease (CAD), with an estimated 30 to 70% of patients displaying hypertension [1–3]. These estimates based on brachial systolic blood pressure (bSBP) underestimate the prevalence of hypertension, as people with normal bSBP could exhibit central systolic pressures (cSBP) equivalent to those with hypertension [4, 5]. Accumulating evidence suggests that cSBP holds greater predictive power for cardiovascular disease compared to bSBP [6–9]. Furthermore, reducing bSBP is a key therapeutic target within cardiac rehabilitation programs [10].

Exploring the acute responses of BP to exercise holds the potential to predict the antihypertensive efficacy of exercise training, potentially elucidating the apparent attenuation of antihypertensive effects in patients with CAD [11]. Investigating acute BP responses becomes pertinent when examining post-exercise hypotension (PEH), a phenomenon characterized by immediate reductions in bSBP following exercise cessation. PEH, often within the range of 8 to 9 mmHg, is a consistent finding in people with and without hypertension [12]; yet, paradoxically, it may be absent in patients with CAD [11, 13, 14]. Despite the acknowledged influence of pre-exercise BP as a moderator of PEH [15], its role in explaining the divergent PEH responses in patients with CAD is dubious, given that patients with and without hypertension exhibit PEH [14, 16]. Alternatively, the substantial inter-individual variability in post-exercise BP responses [12, 17] may be a contributing factor to the conflicting PEH observations in patients with CAD. Previous studies have not investigated individual PEH responsiveness within the CAD context. Moreover, the potential disparate effects of acute exercise on central and brachial BP, akin to those observed with antihypertensive medications [18], remain unexplored. Although PEH of both central and brachial arteries has been reported in patients with CAD [19], a comprehensive understanding of whether acute cSBP responses contribute to the heterogeneous nature of brachial PEH responses is lacking particularly considering effect modifiers. Exercise modifiers, particularly intensity, might also exert influence over post-exercise BP responses. The magnitude of PEH appears to be intensity-dependent, with higher exercise intensities yielding more pronounced reductions in BP in people with and without hypertension (20 to 60-min post-exercise) [20–22]. Considering the association between BP and arterial stiffness, it is conceivable that changes in central arterial stiffness observed after high-intensity exercise could contribute to the apparent intensity-dependent magnitude of PEH [23, 24]. However, whether these findings extend to patients with CAD is unknown, as current evidence was primarily derived from healthy normotensive adults. Furthermore, our understanding of PEH among patients with CAD is limited to aerobic exercise neglecting the relevance of combined exercise regimes which are recommended forms of exercise in cardiac rehabilitation programs.

With these considerations in mind, this study seeks to comprehensively analyze the group and individual central and brachial BP responses at 5, 15 and 30 min post combined exercise of varying intensities (moderate vs high), in participants with and without CAD. As a secondary aim, we examined post-exercise responses of central arterial stiffness. We hypothesized that 1) central rather than brachial BP would be more sensitive to detect PEH in patients with CAD; and that 2) high rather than moderate intensity exercise, would decrease the proportion of non-responders to PEH in patients with CAD.

## Materials and methods

### Study design

This study was designed as a randomized cross-over, repeated measures experiment. All participants underwent two combined exercise sessions of varying intensities, specifically high (HIGH) and moderate (MOD), in a blocked randomized sequence (http://www.randomizer.org/). Before the exercise sessions, all participants underwent both cardiopulmonary exercise testing and 1RM testing, followed by a DEXA scan during a subsequent visit to the laboratory. Each participant completed all experimental sessions consistently at the same time of the day, specifically in the mornings, with at least 48h between sessions to reduce diurnal variation. Post-exercise measurements were conducted at 5, 15 and 30 min after exercise as recommended to assess post-exercise hypotension response [17]. Participants reported to the laboratory in a fasted state (≥ 4h), and refrained from vigorous exercise, vitamin supplements, and foods/beverages containing caffeine and alcohol for at least 12 h preceding each experimental session.

This study was retrospectively registered in ClinicalTrials.gov (NCT06617117) and conducted in strict adherence to the principles outlined in the Declaration of Helsinki and received approval from the Ethical Review Board of the Faculdade de Motricidade Humana–Universidade de Lisboa (02/2018). Although increasingly evident the importance of prospective registration, the policies of the host university only required ethical review board approval and did not promote trial registration. Written informed consent was obtained from all participants who took part in the study. Recruitment took place from 15/03/2018 to 30/03/2020 and the data collection ended on 05-1-2024. There was no follow-up in the present study.

### Participants

**Fig 1:**
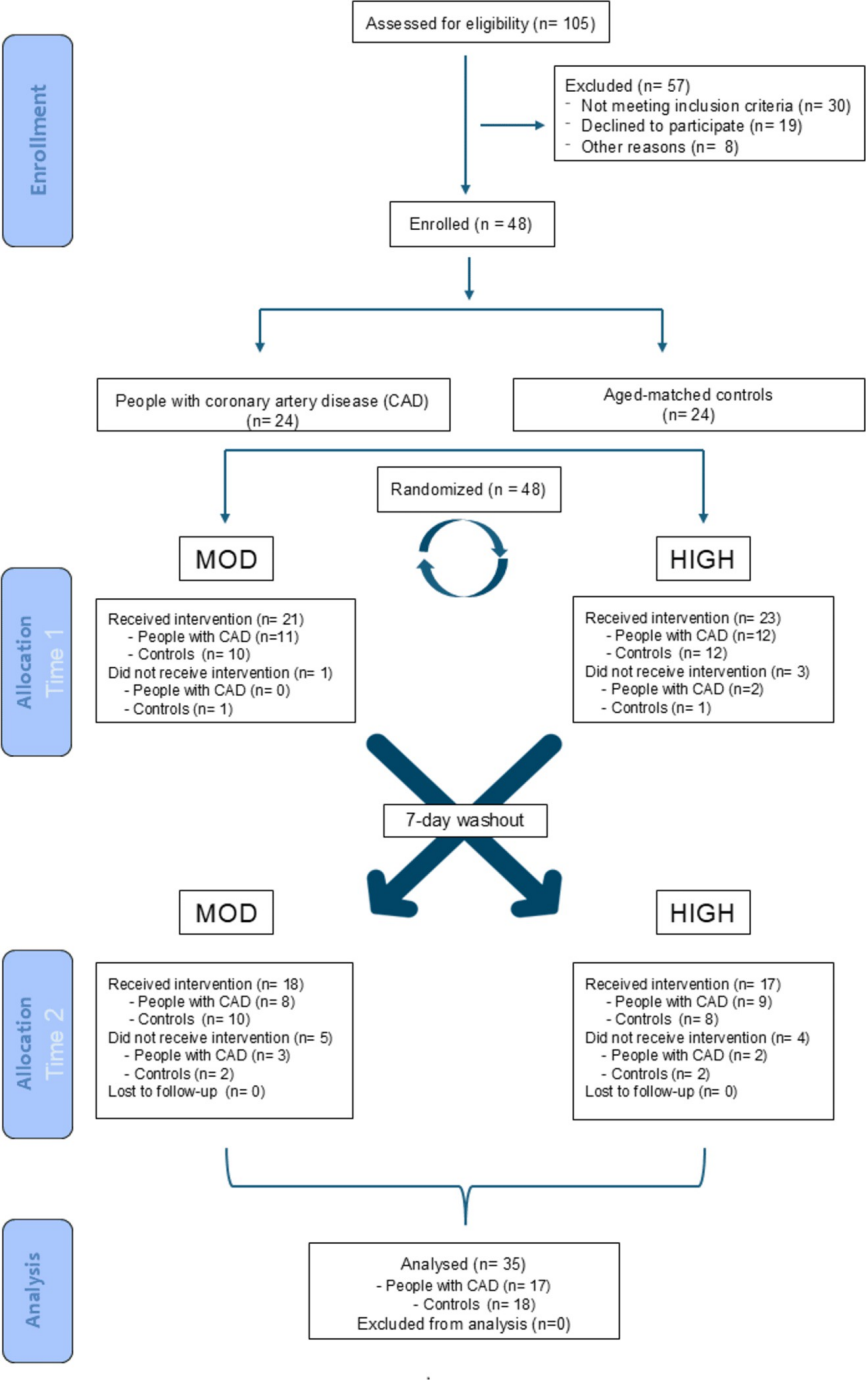
CONSORT flow chart.

Seventeen untrained patients with clinically stable CAD, who were participating in a phase II cardiac rehabilitation program following a recent acute myocardial infarction (within 1–2 months), were recruited to participate in this study (Fig 1). For the control group, 18 apparently healthy, trained participants were recruited from a community gym. These participants had no more than 2 cardiovascular risk factors and had been engaged in exercise at least 3 times per week over the past 6 months. The general exclusion criteria were common to both groups and encompassed cognitive impairment, lung disorders, uncontrolled atrial or ventricular dysrhythmia, disability or mental illness affecting independent decision-making, and extra-cardiac disease affecting physical activity or any other circumstances making participation unsuitable.

### Cardiopulmonary exercise test

All participants underwent a symptom-limited ramp incremental cardiopulmonary exercise test using an electronic bike (Cardiowise Ergoline Xrcise Cycle 1100, Germany). Heart rate (HR) was continuously monitored using a 12-lead EGC, while respiratory gases were collected and analyzed by a metabolic cart (Ergostik, Geratherm Respiratory GmbH, Bad Kissingen, Germany). Brachial BP was assessed by auscultation using an aneroid sphygmomanometer, at rest and every 2 min during the maximal test, while participants were in a seated position. The monitoring of blood pressure and ECG responses was overseen by a physician. Participants were instructed to maintain a cycle cadence of 60–80 rpm with the load increasing by 10–20 W/minute after a 2-minute warm-up period. The exercise test was considered complete when participants met at least 3 of the following criteria: inability to sustain pedaling (>60 rpm) despite encouragement (attributed to volitional and peripheral muscle exhaustion); a respiratory exchange ratio (RER) > 1.10; evidence of a plateau in VO_2_; and a rating perceived exertion > 18. The final phase of the protocol included 2 min of active recovery, involving unloaded pedaling at 50 rpm, followed by 4 min of passive recovery in a seated position. Peak oxygen uptake ($\dot{V}$O_2 peak_) was determined as the highest $\dot{V}$O_2_ attained during the final 30 s of exercise. The first ventilatory threshold (VT1) was identified as 1) the first increase in the ventilatory equivalent for O_2_ (VE/VO_2_), without an increase in the ventilatory equivalent for CO2 (VE/VCO_2_), and 2) the first increase in the expiratory fraction of O_2_. While the second VT as the first increase in the ventilatory equivalent for CO_2_ (VE/VCO_2_) and the first reduction in the expiratory fraction of CO_2_ [25]. Heart rate recovery (HRR1) was calculated as the difference between HR after 1 min of recovery and peak HR.

### Muscle strength testing

The one-repetition (1-RM) method was used to determine the maximal dynamic muscle strength of all participants as described elsewhere [26]. Briefly, 1-RMs were determined for the exercises used in the combined exercise session: Chest Press, Leg Curl, Low Row, Leg Press, Lat Pull-down, and Leg Extension. The protocol comprised four pre-test sessions to familiarize participants with the testing procedures wherein correct exercise and breathing techniques (e.g., avoid the Valsalva maneuver) were taught. Before 1-RM determination for each resistance exercise, all participants warmed up with 8 light repetitions followed by 30-s rest and then completed a second set of 4 moderate repetitions followed by 1-min rest. 1-RM was then determined by instructing participants to perform single repetitions until reaching 1RM. Each attempt was interspersed with a 1-min rest period with the load being increased by 5kg or by 2.5 kg when near the 1-RM.

### Acute combined exercise bouts

The acute bouts of combined exercise adhered to the current cardiac exercise rehabilitation guidelines [27]. These sessions were structured as follows: each session lasted 1h and was divided into a warm-up (10 min), aerobic exercise (20 min), followed by a circuit resistance exercise (20 min), and concluded with a cool-down phase involving passive stretching for 10 min. The warm-up in both bouts included cycling with an intensity 10% above the first ventilatory threshold (VT1). In the moderate-intensity bout, the main segment consisted of an 18-min cycling continuous aerobic exercise at VT1, followed by 2-min of unloading cycling. Subsequently, participants engaged in resistance exercises ‐ 2 sets of 12 repetitions at 60% of their 1RM. These resistance exercises were part of a machine-assisted circuit training program, encompassing Chest Press, Leg Curl, Low Row, Leg Press, Lat Pull-down, and Leg Extension. In the high-intensity bout, the main segment involved interval cycling exercise, comprising 5x2 min at VT2 interspersed with 4x2 min at VT1, followed by 2-min de-loading. Subsequently, participants completed resistance exercises (2 sets of 12 repetitions), targeting the same muscle groups, at an intensity of 80% of their 1RM. An exercise physiologist closely supervised aerobic exercise intensity using a heart rate monitor and the talk test, resistance load selection and instructed participants to maintain a 60–65 rpm for the aerobic exercise and to avoid the Valsalva maneuver during resistance exercise. The cool-down in both bouts consisted of 20-s of passive stretching.

To quantify the combined exercise load, we employed the training impulse method (TRIMP), which encompasses the sum of aerobic load (i.e., time spent in each heart rate training zone), and resistance load (i.e., number of sets x number of repetitions x 1-RM) [28]. The TRIMP scores were similar between moderate- (TRIMP = 38 +1800), and high-intensity- (TRIMP = 48 + 1600) combined exercise bouts.

### Blood pressure and central arterial stiffness

All measurements were conducted with participants supine in a climate (22°C), dim-light controlled room with baseline measurements preceded by a 10-min rest period. cSBP was measured using non-invasive carotid tonometry (Complior, ALAM Medical, Paris, France). Carotid waveforms obtained with a piezoelectric sensor were calibrated from brachial diastolic blood pressure (bDBP) and mean arterial pressure (i.e., 2/3 bDBP + 1/3 bSBP), which were assumed to remain constant throughout the vascular system [29, 30]. To ensure measurement reliability, a single operator with more than 100 hours of tonometry conducted 2 repeated measurements on the right side of the body, each consisting of 10 waveforms with quality indexes >90%. The average of the two measurements were used for data analysis. The added pressure arriving from wave reflection on SBP was derived from tonometry measurements as the difference between bSBP and cSBP. In our participants with CAD, the coefficients of variation of cSBP and bSBP measurements were 5%, while in controls were 4%. Brachial BP was measured in the supine position using an Omron automatic sphygmomanometer (HEM-907-E Omron Health Care, Kyoto, Japan). Hypertension was defined according to the ESH guidelines (i.e., bSBP ≥ 140 mmHg and/or bDBP ≥ 90 mmHg). Amplification pressure was defined as the brachial minus central systolic pressure.

We also assessed central arterial stiffness via carotid-femoral pulse wave velocity (cf PWV), wherein both carotid and femoral waveforms were collected simultaneously using piezoelectric pressure mechanotransducers (Complior, ALAM Medical, Paris, France). Pulse transit times (PTT) were automatically calculated using the intersect tangent algorithm of the foot-to-foot method, enabling the calculation of cfPWV as the ratio of distance to PTT. Travel time distances were defined as the taped measured distance over body surfaces between the two recording sites of interest, with the cf distance corrected by a factor of 0.8. The research team involved in the data collection reviewed all raw data on the same day of the visit. To ensure data integrity, biweekly examinations were conducted by the corresponding author.

### Individual blood pressure responsiveness to acute combined exercise

Participants were classified as either showing relevant (responder) or negligible (non-responder) post-exercise hypotensive or hypertensive responses using the ROPE + HDI decision rule to reject or non-reject the null, a Bayesian method [31, 32]. Briefly, this method estimates the percentage of the highest density interval (HDI, similar to the confidence intervals in frequentist statistics) within the range of values around the null–region of practical equivalence (ROPE). This estimated percentage corresponds to different levels of significance, which guided the classification of post-exercise hypotension or hypertensive response, with non-responders defined as > 99% of HDI and responders as < 1% within the ROPE [31, 32]. We also considered an undecided category [% of HDI inside ROPE: ≤ 98% to ≥ 2%].

Both the HDI and the ROPE were computed with the R package bayestestR [33]. The HDI was calculated as an 89% credible interval and derived from each participant’s posterior normal distribution obtained from 1000 simulations based on the individual post-exercise changes (delta) in cSBP and bSBP (both 15 and 30-min) [31, 32, 34]. Individual normal distributions were derived using the R’s rnorm function. The standard deviation of each distribution was defined as the individual TE * √2, where TE is the technical error calculated as the coefficient of variation * baseline mean of cSBP and bSBP) (Swinton et al. 2018). The coefficient variation used for both cSBP and bSBP are reported in the section ‐ Blood pressure and central arterial stiffness. We estimated the ROPE as 20% of the baseline cSBP and bSBP standard deviation in each group (ROPE; cSBP: CAD, -3.60 to 3.60 mmHg; CON: -3.20 to 3.20 mmHg; bSBP: CAD: -3.71 to 3.71 mmHg, CON: -3.24 to 3.24 mmHg) [35]. The ROPE represents the smallest worthwhile difference [31, 35, 36]. Despite the lack of consensus on how to define a true post-exercise BP response, a methodologically sound approach is that BP changes should surpass the technical error of measurement [17].

### Statistics

All statistical analyses were conducted using R software, version 4.3.2 [37], with a significant level (α) set at 0.05. Data are presented as mean (SD) unless otherwise stated. We assessed the normality and homogeneity of residuals of all models using the Shapiro-Wilk and Breusch–Pagan tests, respectively. We also inspected QQ plots using the R performance package [38]. Participant characteristics were compared using Welch’s independent-sample *t*-tests for numerical outcomes and Fisher’s exact tests for categorical outcomes.

To analyse post-exercise changes in BP and central arterial stiffness (i.e., cfPWV), we used linear mixed models fitted with restricted maximum likelihood. We applied Satterthwaite’s method to approximate degrees of freedom for the F test from the lmerTest package [39]. The fixed effects in the models included time, condition, and group, while each participant was assigned as a random intercept. We calculated partial omega squares (*ω*^*2*^) for main effects and interactions (intensity-by-time; group-by-time; group-by-condition and intensity-by-time-by-group) using sjstats package and interpreted them based on Cohen’s [40] benchmarks [small (ω^*2*^ < 0.05), medium (ω^*2*^ < 0.25), and large ω^*2*^ > 0.25) effects sizes]. The linear mixed models were adjusted for potential confounders, including medication, $\dot{V}$O_2_ peak, HR_peak_, and HR at each time point. Cofounders were entered one at a time, as a covariate, in the model to evaluate their individual contributions. Post-hoc comparisons were performed using the Bonferroni test with the emmeans package when significant main effects and interactions were present. Effect sizes (i.e., Hedges’ *g*) were also computed for post-hoc comparisons as the mean contrast divided by the pooled standard deviation, using the emmeans package *[*Cohen’s benchmarks [40]: small (g < 0.2), medium (*g*; 0.2–0.5), and large (*g*: 0.5–0.8)*]*.

## Results

### Characteristics of the participants

The clinical and demographic characteristics of participants are presented in Table 1. Participants with CAD exhibited lower $\dot{V}$O_2 peak_ and HR_max_ in comparison to the control group (difference (d) = -7.91 ml.kg.min^-1^, 95% CI: -11.81 to -4.00 ml.kg.min^-1^, *p* < 0.001; d = -14 b.min^-^, 95% CI: -27 to -1 b.min^-1^, *p* = 0.039). A higher proportion of participants with CAD had hypertension and were under angiotensin-converting enzyme inhibitors (ACE), β blockers, statins, and anticoagulant medication, as compared to the control group (Table 1).

**Table 1:** Characteristics of the participants.

	CAD (n = 17)	CON (n = 18)	*p*-value
Age (years)	63 (8)	62 (7)	0.673
Sex (counts, M/F)	13/4	14/4	0.999
Height (m)	1.66 (0.09)	1.68 (0.09)	0.527
Body mass (kg)	73.75 (12.62)	73.51 (15.74)	0.961
BMI (kg.m^-2^)	26.65 (3.18)	25.79 (4.01)	0.521
Body fat mass (%)	30.19 (5.07)	29.72 (4.59)	0.778
cSBP (mmHg)	101 (18)	122 (18)	0.002
bSBP (mmHg)	112 (8)	122 (9)	0.002
Amplification, bSBP–cSBP (mmHg)	9 (17)	0 (14)	0.039
bDBP (mmHg)	70 (6)	77 (7)	0.003
cPP (mmHg)	32 (21)	46 (20)	0.052
Resting HR (b.min^-1^)	63 (9)	63 (11)	0.985
HR_max_ (b.min^-1^)	130 (20)	144 (20)	0.039
HRR1 (b.min^-1^)	18 (8)	16 (6)	0.644
$\dot{V}$O_2 peak_ (ml.kg.min^-1^)	21.95 (4.91)	29.86 (6.37)	<0.001
*Medication (counts yes/no)*			
ACE inhibitors	8/9	2/16	0.027
β blockers	14/3	0/18	<0.001
Ca^2+^ channel blockers	2/15	0/18	0.229
Anticoagulants	16/1	0/18	<0.001
Statins	16/1	13/5	<0.001
*Stage 1 Hypertension (counts*, *yes/no)*	11/6	5/13	0.044

Continuous data are presented as mean (SD). Medication data are presented as counts of non-taking / and taking medication. *p*-values of Welch two Sample t-test and Fisher exact test are presented for continuous and categorical data, respectively. Abbreviations: BMI, body mass index; cSBP, central systolic blood pressure, bSBP, brachial systolic blood pressure, bDBP, brachial diastolic blood pressure $\dot{V}$O_2,_ oxygen uptake; HRR1, heart rate recovery after 1 min of maximal aerobic exercise testing, ACE, angiotensin-converting enzyme; Ca^2+^, calcium.

### Resting blood pressure and central arterial stiffness

Participants with CAD displayed lower cSBP (d = -21 mmHg, 95% CI: -32 to -8 mmHg, *p* = 0.002, *g* = -1.31), bSBP (d = -10 mmHg, 95% CI: -16 to -4 mmHg, *p* = 0.002, g = -1.25) and bDBP (d = -7 mmHg, 95% CI: -12 to -3 mmHg, *p* = 0.003, *g* = -1.48) than the control group. Participants with CAD exhibited amplification pressure but it was absent in controls (d = 10 mmHg, 95% 1 to 21 mmHg, *p* = 0.039, *g =* 0.68, Table 1). cfPWV did not differ between groups (*p* = 0.708, *g* = -0.24).

### Post-combined exercise blood pressure and central arterial stiffness

Group-by-time interaction effects were observed for cSBP [F(3, 229) = 16.64, *p* < 0.001, ω^2^ = 0.16] and bSBP [F(3, 229) = 4.40, *p* = 0.003, ω^2^ = 0.05, Table 2]. Participants with CAD exhibited higher cSBP increases 5-min after both HIGH and MOD (d_5-bas_ = 16 mmHg, 95% CI: 10 to 23 mmHg, *p* < 0.001, *g* = 1.10) compared to those without CAD (d_5-bas_ = 7 mmHg, 95% CI: 1 to 14 mmHg, *p* < 0.001, g = 0.49). A group-by-time-condition was noted bSBP (Table 2), which indicated increases only in the control group to a larger extent 5-min after MOD (d_5-bas_ = 12 mmHg; 95% CI: 3 to 19 mmHg, *p* < 0.001, g = -1.23). Post-exercise hypotensive effects were observed 15-min into recovery only in participants without CAD for cSBP (d_15-rest_ = -10 mmHg, 95% CI: -16 to -4 mmHg, *p* = 0.003, *g* = -0.60), but not for bSBP (d_15-bas_ = -4 mmHg, 95% CI: -2 to 8 mmHg, *p* = 0.999, *g* = 0.35). This central hypotensive effect persisted 30-min into recovery (Table 2). Conversely, participants with CAD still had elevated cSBP 30-min into recovery compared to baseline (d_30-bas_ = 10, 95% CI: 4 to 17 mmHg, *p* = 0.001, *g* = 0.64). A main effect of time was noted for bDBP (Table 2), indicating PEH 15- min into recovery (d_15-bas_ = -3 mmHg, 95% CI: - 6 to -1 mmHg, *p* = 0.046, *g* = -0.40), which returned to baseline levels at 30-min post-exercise. Amplification pressure 30 min into recovery was abolished in participants with CAD (d_30-rest_ = -10 mmHg, 95% CI: -18 to -4 mmHg, *p* = 0.002, *g* = -1.01), while it was increased in controls (d30-rest = 7 mmHg, 95% CI: 1 to 14 mmHg, p = 0.028, *g* = 0.52) Model adjustments for HR at each time point, cSBP and bSBP baseline values, $\dot{V}$O_2 peak_ and medication did not change the results.

**Table 2:** Group-mean blood pressure responses to acute combined exercise of different intensities.

	CAD		CON		*Time*	*Intensity*	*Group*	Time*Intensity	Group*Time	Group*Time*Intensity
	MOD	HIGH	MOD	HIGH	*p (*ω^*2*^*)*	*p (*ω^*2*^*)*	*p (*ω^*2*^*)*	*p (*ω^*2*^*)*		
HR (bpm)					**< 0.001 (0.25)**	0.197 (0.00)	0.362 (0.00)	0.806 (0.00)	0.440 (0.00)	0.963 (0.00)
Pre	60 (11)	63 (12)	63 (18)	64 (18)						
Post 5	69 (10)*	72 (10)*	75 (12)*	76 (9)*						
Post 15	67 (10)*	68 (10)*	71 (12)*	70 (10)*						
Post 30	67 (11)*	66 (11)*	68 (11)*	68 (10)*						
cSBP (mmHg)					**< 0.001 (0.19)**	0.667 (0.00)	**0.172 (0.03)**	0.990 (0.00)	**< 0.001 (0.16)**	0.880 (0.00)
Pre	101 (18)	103 (18)	122 (18)	120 (16)						
Post 5	118 (13)*	119 (15)*	128 (18)*	129 (20)*						
Post 15	110 (14)*ⱡ	113 (13)*ⱡ	114 (13)*ⱡ	110 (9)*ⱡ						
Post 30	111 (13)*ⱡ	114 (11)*ⱡ	111 (12)*ⱡ	107 (10)*ⱡ						
bSBP (mmHg)					**< 0.001 (0.17)**	0.187 (0.00)	**0.004 (0.21)**	0.576 (0.00)	**0.003 (0.05)**	**0.028 (0.03)**
Pre	112 (8)	112 (6)	122 (8)	121 (9)						
Post 5	113 (9)	118 (12)	134 (17)*	124 (10)						
Post 15	113 (9)	113 (12)	121 (11)	116 (9)						
Post 30	112 (10)	111 (14)	118 (10)	116 (9)						
*Amplification bSBP-cSBP (mmHg)*										
Pre	11 (17)	9 (16)	0 (14)	1 (14)	0.001 (0.05)	0.540 (0.00)	0.399 (0.00)	0.720 (0.00)	**< 0.001 (0.08)**	0.084 (0.02)
Post 5	-5 (13)*	-1 (12)*	6 (8)	-5 (19)						
Post 15	4 (13)*	-1 (17)	7 (6)*	6 (6)*						
Post 30	1 (10)*	-3 (14)*	7 (5)*	9 (9)*						
*bDBP (mmHg)*					**0.008** (0.04)	0.62 (0.00)	**0.012 (0.16)**	0. 671 (0.00)	**0.015 (0.03)**	0.985 (0.00)
Pre	70 (6)	71 (5)	77 (7)	78 (7)						
Post 5	72 (5)	72 (6)	76 (7)	76 (8)						
Post 15	70 (5)	67 (17)	73 (8) *ⱡ	74 (8) *ⱡ						
Post 30	71 (6)	70 (7)	73 (8)*ⱡ	72 (7)*ⱡ						
*bPP (mmHg)*										
Pre	40 (9)	41 (8)	45 (7)	43 (8)	**< 0.001 (0.10)**	0.120 (0.00)	0.081 (0.06)	0.991 (0.00)	**<0.001 (0.17)**	0.841 (0.00)
Post 5	41 (8)	46 (10)	58 (15)*	48 (10)*						
Post 15	43 (7)	46 (9)	48 (9)	42 (8)						
Post 30	41 (7)	41 (8)	45 (8)	44 (9)						
*cPP (mmHg)*					**< 0.001 (0.13)**	0.738 (0.00)	0.620 (0.00)	0.988 (0.00)	**< 0.001 (0.08)**	0.854 (0.00)
Pre	32 (21)	32 (22)	45 (17)	47 (21)						
Post 5	45 (14)*	47 (16)*	53 (15)	51 (23)						
Post 15	39 (16)*	43 (17)*	41 (12)*	34 (12)*						
Post 30	39 (12)*	44 (15)*	38 (11)*	35 (10)*						
cfPWV (m.s^-1^)										
Pre	8.11 (1.61)	8.29 (1.65)	8.38 (1.76)	8.50 (1.82)	**0.010 (0.04)**	0.464 (0.00)	0.708 (0.00)	0.841 (0.00)	0.300 (0.00)	0.230 (0.00)
Post5	8.47 (1.56)	8.72 (1.56)	9.00 (1.81)	9.09 (2.37)						
Post 15	8.58 (1.36)	8.29 (1.16)	8.01 (1.72)	8.66 (1.74)						
Post 30	8.51 (1.37)	8.15 (1.36)	8.50 (1.77)	8.77 (1.87)						

Data presented as mean (SD). Abbreviations; HR, heart rate; cSBP, central systolic blood pressure; bSBP, brachial systolic blood pressure; bDBP, brachial diastolic blood pressure; bPP, brachial pulse pressure; cPP, central pulse pressure; cfPWV, carotid-femoral pulse wave velocity.

* Different from pre (*p* < 0.01)

ⱡ Different from post-5 min (*p* < 0.01), Only significant interactions are reported

A main effect of time was observed for cfPWV (Table 2), which indicated increases in cfPWV (d = 0.46, 95% CI: 0.05 to 0.87 m.s^-1^, *p* = 0.018, *g* = 0.52) at 5-min post-exercise returning to baseline 30-min into recovery. Adjustments for HR at each time point, HR_max_, $\dot{V}$O_2 peak,_ and medication did not change above central arterial stiffness inferences. However, the time effect was abolished when adjusting the cfPWV model for cSBP and bSBP.

### Blood pressure individual responsiveness to acute combined exercise

**Fig 2:**
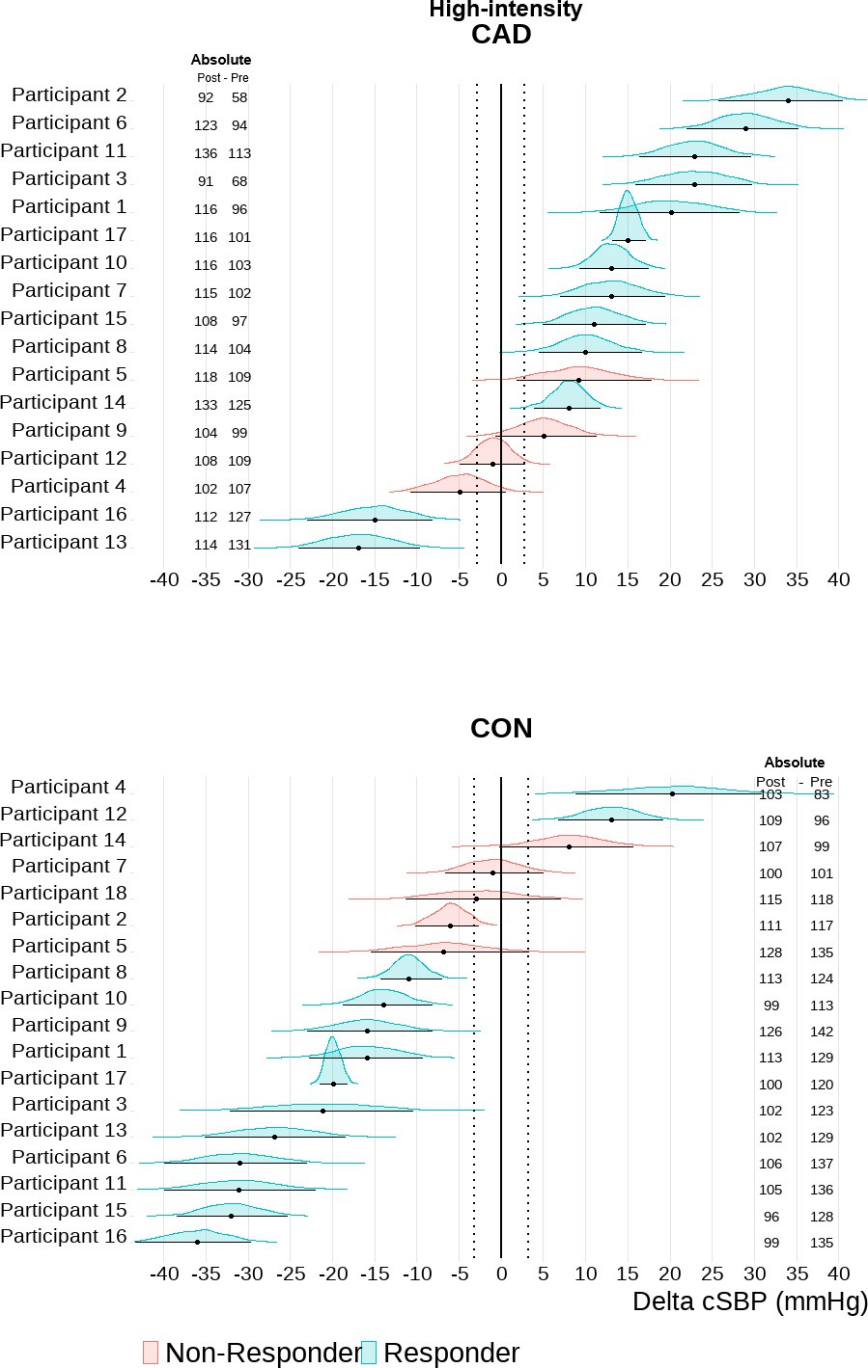
Central systolic blood pressure (cSBP) individual responsiveness after high-intensity combined exercise in participants with coronary artery disease (CAD, n = 17) and controls (CON, n = 18). Individual response analyses are based on the percentage of the highest density (error bars of each individual distribution) within the range of values around the null–region of practical equivalence (ROPE). CAD, -3.60 to 3.60 mmHg; CON: -3.20 to 3.20 (i.e., represented by the vertical dotted lines). Absolute values are also depicted for each participant as post 30 min and pre cSBP values.

**Fig 3:**
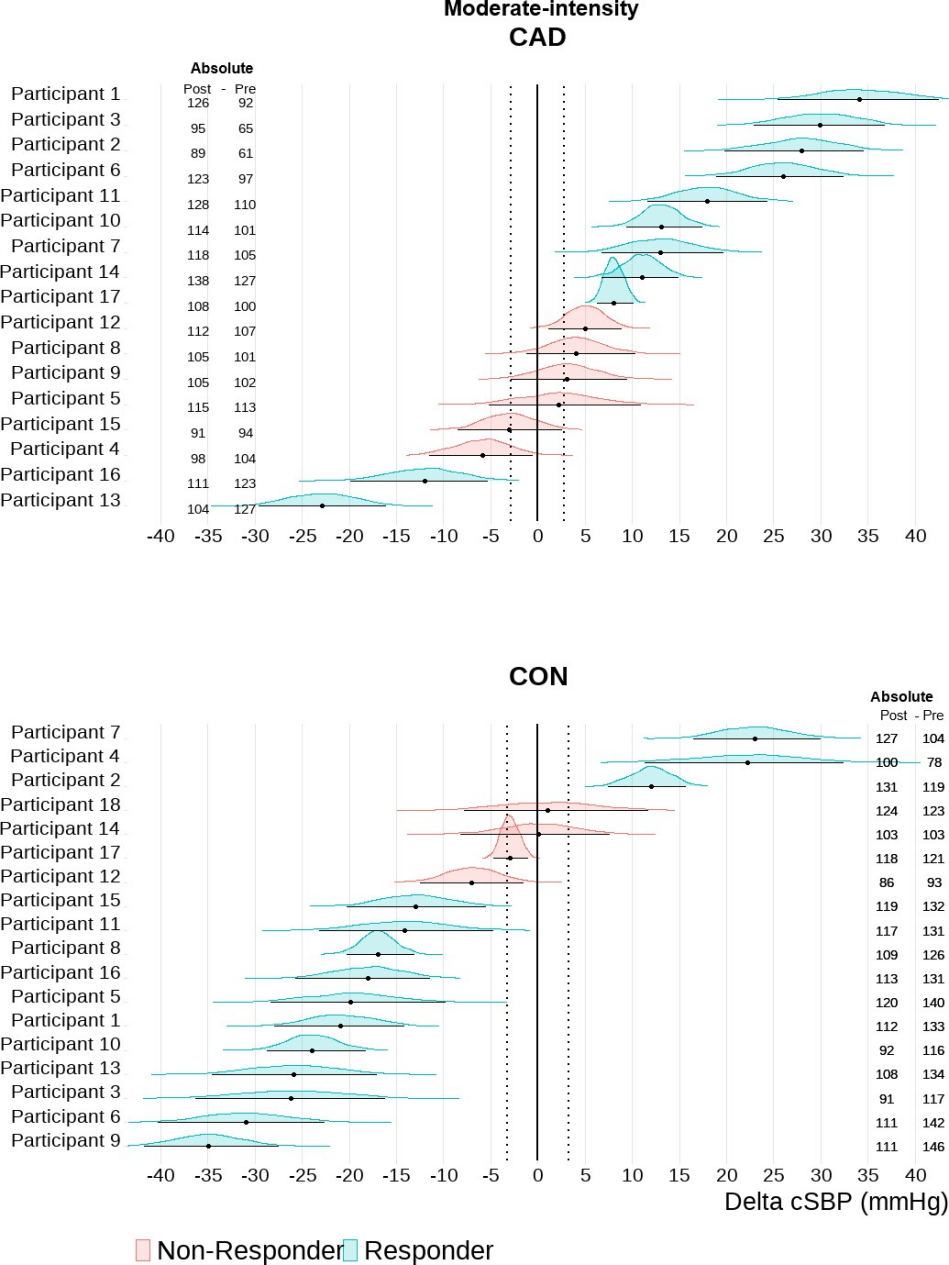
Central systolic blood pressure (cSBP) individual responsiveness after moderate-intensity combined exercise in participants with coronary artery disease (CAD, n = 17) and controls (CON, n = 18). Individual response analyses are based on the percentage of the highest density (error bars of each individual distribution) within the range of values around the null–region of practical equivalence (ROPE). CAD, -3.60 to 3.60 mmHg; CON: -3.20 to 3.20 (i.e., represented by the vertical dotted lines). Absolute values are also depicted for each participant as post 30 min and pre cSBP values.

**Fig 4:**
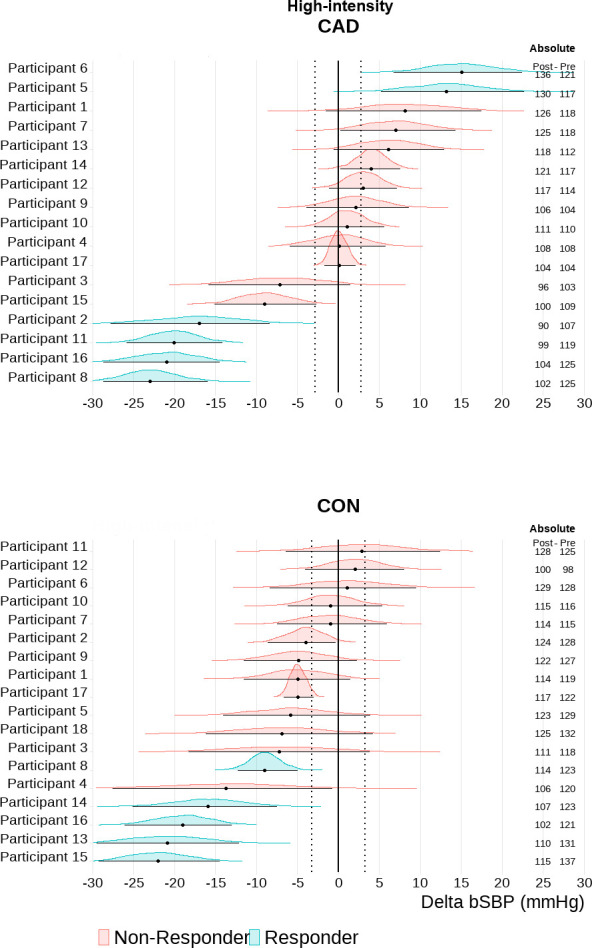
Brachial systolic blood pressure (bSBP) individual responsiveness after high-intensity combined exercise in participants with coronary artery disease (CAD, n = 17) and controls (CON, n = 18). Individual response analyses are based on the percentage of the highest density (error bars of each individual distribution) within the range of values around the null–region of practical equivalence (ROPE); (CAD: -3.71 to 3.71 mmHg, CON: -3.24 to 3.24 mmHg, i.e., represented by the vertical dotted lines). Absolute values are also depicted for each participant as post-30 min and pre cSBP values.

**Fig 5:**
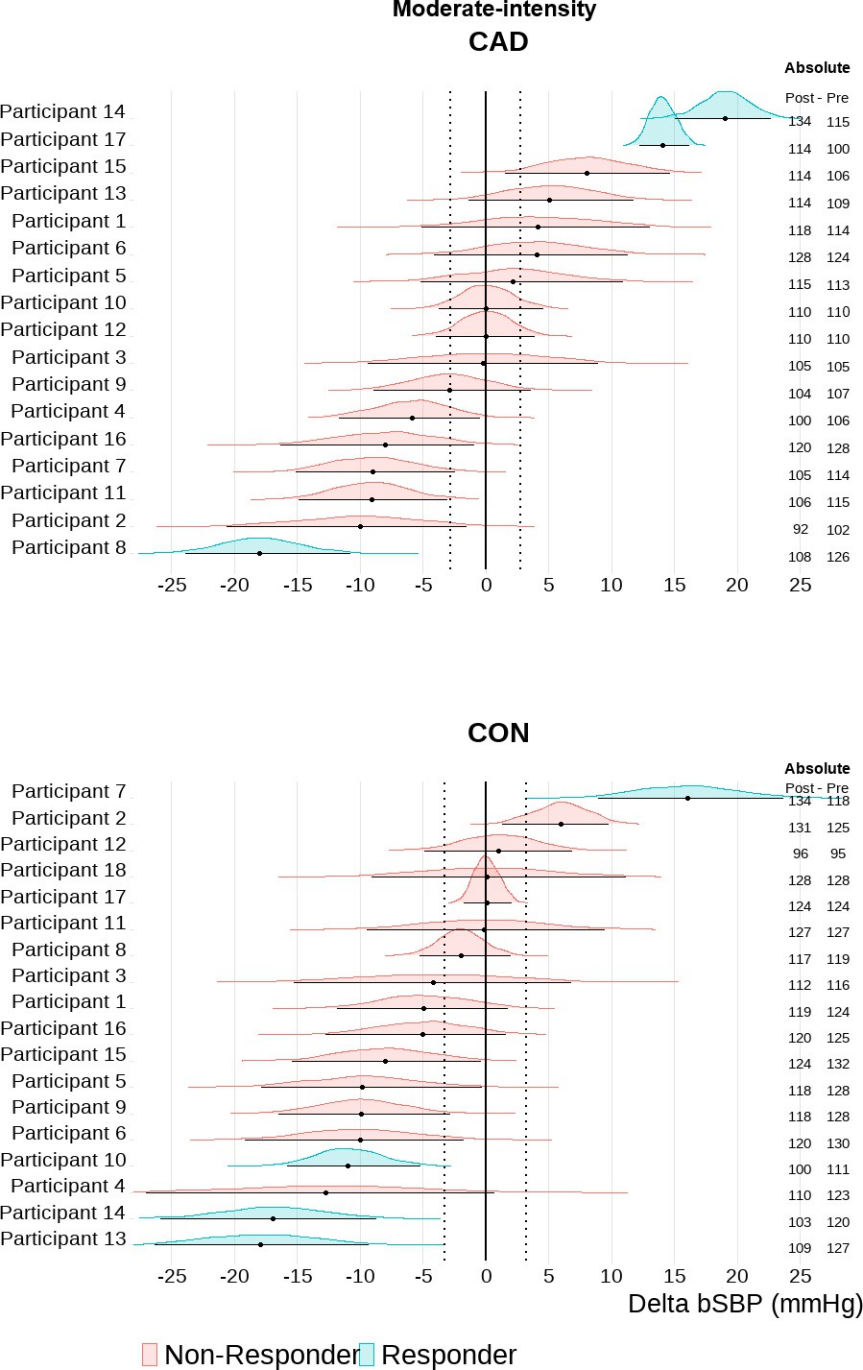
Brachial systolic blood pressure (bSBP) individual responsiveness after moderate-intensity combined exercise in participants with coronary artery disease (CAD, n = 17) and controls (CON, n = 18). Individual response analyses are based on the percentage of the highest density (error bars of each individual distribution) within the range of values around the null–region of practical equivalence (ROPE); (CAD: -3.71 to 3.71 mmHg, CON: -3.24 to 3.24 mmHg, i.e., represented by the vertical dotted lines). Absolute values are also depicted for each participant as post 30 min and pre cSBP values.

Based on an individual TE response threshold, the majority of participants with CAD showed sustained elevations in cSBP 30-min into recovery after exercise [HIGH: n = 11 (65%); MOD: n = 9 (53%] (Figs 2 and 3). Only 2 participants with CAD (12%) showed a central hypotensive response after both HIGH and MOD. The majority of controls exhibited a central hypotensive response after exercise [HIGH: n = 11 (61%); MOD: n = 11 (61%)] (Figs 2 and 3). Elevated cSBP was observed in 3 (17%) controls after MOD and 2 (11%) after HIGH. Additionally, 4 (22%) and 5 (28%) controls were non-responders in MOD and HIGH, respectively. bSBP exhibited greater variability in participants with and without CAD (Fig 4). bSBP post-exercise responsiveness in participants with CAD was characterized by a high number of non-responders both in MOD [n = 14 (82%)] and HIGH [n = 11 (65%)] (Figs 4 and 5). Brachial hypotensive responses were only observed in 1 (6%) and 4 (24%) participants with CAD after MOD and HIGH, respectively. In the control group, most participants were post-exercise bSBP non-responders [MOD = 14 (78%) vs HIGH = 13 (72%)] (Figs 4 and 5). Brachial hypotensive responders were observed only in 3 (17%) and 5 (28%) controls in MOD and HIGH, respectively. None of the participants with and without CAD fell under the category “undecided”.

## Discussion

While it is well established that brachial pressure, due to systolic pressure amplification, does not accurately reflect central aortic pressure, this study is the first to examine the post-exercise effects on central and brachial BP, specifically in patients with CAD. Our findings reveal a persistent elevation in cSBP in patients with CAD, regardless of combined exercise intensity, contrasting with controls who exhibited PEH during early recovery (15 and 30-min). Conversely, bSBP remained unaffected by combined exercise in patients with CAD, while controls experienced a transient post-exercise elevation that normalized within 15-min of recovery. The individual post-exercise responses consistently mirrored these overall patterns, with a similar number of “responders” for both HIGH and MOD. These results challenge our initial hypothesis and suggest that central blood pressure provides different, and potentially more relevant information than brachial BP in patients with CAD. However, whether this difference impacts risk assessment remains to be determined.

### Resting blood pressure

Although participants with CAD had a higher prevalence of hypertension, their central and brachial BP readings were lower compared to controls, within the normotensive range (brachial: <120/80 mmHg, cSBP <112 mmHg). This indicates effective BP control via medication attributed to combined pharmacotherapy, including ACE inhibitors and beta-blockers, which effectively reduce both central and brachial pressures [41]. In addition, many participants with CAD (n = 13) were on vasodilating beta-blockers shown to have greater central rather than brachial hypotensive effects, contributing to the reported preservation of pressure amplification [42–44]. In line with the normal BP, stiffening of the central arteries was not observed, which together suggests adequate coronary perfusion pressure and ventricular afterload in patients with CAD–key to preventing pulsatile organ damage [45–47].

### Post-combined exercise blood pressure response

#### Central blood pressure

During the early recovery phase, both group and individual analyses confirmed that participants with CAD exhibited a sustained elevation in cSBP, contrasting to the hypotensive response observed in controls at 15- and 30-min post-exercise. Contrary to our findings, Hintsala et al. [19] reported central PEH to aerobic exercise, such discrepancy may relate to differences in resting central BP (i.e., lower in our study). While both our groups showed minor central arterial stiffening, which likely contributed to increased cardiac output and pulsatile pressure across the arterial tree, this did not account for the contrasting central BP. In fact, post-exercise central BP and arterial stiffness responses have been found to be unlinked in clinical populations, but the culprit mechanism remains unknown [48]. PEH mechanisms, including histamine receptor activation (i.e., H_1_ and H_2_) and baroreflex resetting have been suggested to contribute to the central PEH effect observed in controls [49, 50]. In fact, the systolic augmentation pressure due to wave reflection normalized in controls during early recovery, possibly due to peripheral vasodilation, but not in patients with CAD. The vasoconstrictor role of histamine in atherosclerotic coronary vessels and its elevation in CAD patients raise questions about its post-exercise effects in patients with CAD [51, 52]. Recent findings indicate redundancy in BP regulation via histamine, as shown in unaltered exercise hyperemia and post-exercise central BP after histamine receptor blockade in healthy adults [53, 54]. Baroreflex resetting may not explain the post-exercise differential central BP response in participants with CAD and controls as both exhibited similar post-exercise changes in HR (the modulated variable of the parasympathetic arm), and DBP (the regulated variable of the sympathetic arm) [55]. Additionally, differences in baroreceptor stretch-induced transduction are unlikely to underline the differential central pressure response after exercise, as central arterial stiffness was similar across groups. Thus, the mechanism behind the differential post-exercise central BP response noted remains to be determined.

#### Brachial blood pressure

Both participants with and without CAD showed no significant post-exercise changes in bSBP, suggesting that central BP and bDBP (e.g., small PEH effect ∼3 mmHg) are more responsive to exercise-induced vasomotor and wave reflection changes than bSBP. These findings contrast with reports supporting a sustained PEH response up to 24h in patients with CAD [11, 13, 14]. This intricate post-exercise brachial BP response in patients with CAD results from a multifaceted interplay of biological, methodological and exercise factors.

From a biological standpoint, individuals with higher pre-exercise BP tend to exhibit more pronounced PEH, a phenomenon seen in both patients with hypertension and CAD [14, 16]. Although our participants with CAD had lower resting BP than those without CAD, both groups showed null brachial systolic PEH responses, with baseline adjustments having no impact on the outcomes. Furthermore, while individual variability often underlies null group mean changes in post-exercise BP in patients with peripheral artery disease (47), our study showed that only a subset of patients with CAD (only 5 to 6) and approximately half of the control group demonstrated brachial systolic PEH.

Timing of post-exercise BP measurements and position are also important methodological factors to consider in the highly variable post-exercise BP response. For example, Iellamo et al. [14] reported hypotensive effects 60-min after exercise in patients with CAD, whereas Fagard & Vanhees [13] also failed to detect PEH within 20 and 60-min after moderate exercise intensity. Differences in BP measurements between seated and supine positions can influence PEH, as the seated position may increase blood pooling in the legs, thus exaggerating any BP changes over time [56]. However, it is important to note that our study, like the research conducted by Iellamo et al. [14], assessed BP in the supine position, and we did not observe brachial systolic PEH.

Combined exercise of high-intensity failed to increase the number of brachial BP responders among groups. Such finding suggests that intensity is not a key modulator of post-exercise BP response to combined as it is for aerobic exercise [20–22]. Exercise modality has been recently acknowledged as an important confounding factor behind the large variability of post-exercise BP response [57]. However, it is unlikely that combined exercise *per se* prevented brachial systolic hypotensive effects, as PEH has been documented in patients with CAD [16]. In fact, to date, none of the common exercise modalities has shown evidence of greater BP lowering acute effect (Pescatello et al. 2019) [58], and combined exercise is recommended in cardiac rehabilitation settings. Future investigations on post-exercise BP responses to exercise, considering both central and brachial pressure and the interplay between exercise intensity and modality, are warranted to shed light on the present mixed literature.

### Relevance of central BP post-exercise changes in CAD

Persistent elevations and delayed recovery of bSBP after acute exercise are associated with an increased risk for hypertension, myocardial infarction, cardiovascular disease, and all-cause mortality [59, 60]. Although bSPB remained unchanged in participants with CAD, their sustained elevation in cSBP during recovery, with most exhibiting increments >5 mmHg, may predispose to a transient insult to vital organs, including the heart, brain, and kidneys, as these organs are exposed to central rather than brachial BP [5, 61]. However, both the clinical relevance of the sustained elevation of cSBP in patients in CAD and whether cSBP during exercise recovery holds greater predictive value than bSBP for cardiovascular events and disease remains to be determined.

### Limitations

This study is not without limitations. First, the absence of measurements of baroreflex sensitivity, muscle sympathetic nerve activity, plasma catecholamines and histamine concentrations precludes a more in-depth understanding of mechanistic aspects underlying the regulation of central and brachial BP after combined exercise. Second, the timing of our BP assessments during the early recovery phase may not have captured the entirety of PEH, particularly in the case of bSBP, which can manifest beyond the observed time frame (e.g., 1–24 hours). Third, the use of the 24h ambulatory BP monitoring could have provided valuable insights into the residual and dipping effects of acute combined exercise on BP. Fourth, the widespread use of anti-hypertensive medication among participants with CAD may have introduced confounding factors, particularly after combined exercise. This is noteworthy given that some medications, such as ACE inhibitors, are known to blunt brachial PEH [62]. While it would have been ideal to temporarily suspend medication use for study purposes, ethical and medical considerations took precedence. Lastly, our findings (i.e., *p*-values) must be interpreted with caution given that we used a small convenience sample, which may have resulted in limited statistical power, particularly concerning the assessment of bSBP. Still, our sample was sufficient to detect moderate unbiased effect sizes for the group-by-time interaction of our main outcomes (i.e., cSBP and bSBP).

## Conclusions

Our results revealed distinct post-exercise effects on central and brachial BP, which were consistent across different exercise intensities but not specific to the clinical population. Notably, this study is the first to demonstrate that participants with CAD exhibit a persistent elevation in central BP response following exercise, while controls exhibited a hypotensive response. Surprisingly, both groups showed an unchanged bSBP at 15 and 30-min into recovery. Individual response analyses confirmed group post-exercise trends of both cSBP and bSBP in people with CAD and controls. These findings indicate that post-exercise central BP provides different and potentially more informative data than brachial BP in patients with CAD, though its clinical relevance remains to be determined.

## Supporting information

S1 File Dataset of the current study. (XLSX)

S2 File Research plan approved by Ethical Review Board of the Faculdade de Motricidade Humana–Universidade de Lisboa (02/2018). (DOCX)

S3 File CONSORT checklist. (DOC)
