# Supplementary material for: Post-exercise differential response of central and brachial blood pressure in patients with coronary artery disease: A randomized crossover trial
Source: PLoS One. 2025 Feb 21;20(2):e0317212. doi: 10.1371/journal.pone.0317212 (PMC11844900; doi:10.1371/journal.pone.0317212)
Supplement: S2 File — (DOCX) [file pone.0317212.s002.docx]

Research plan

## Título do estudo ou projeto (designação, em português e inglês, a utilizar pelo CEFMH)

The acute dynamic responses of different exercise intensity-dependent on autonomic and vascular systems in coronary artery disease patients. Respostas agudas dinâmicas de diferentes exercícios de intensidade-dependente nos sistemas autónomo e vascular em pessoas com doença das artérias coronárias.

## Nome do proponente (pessoa responsável pelo estudo para o CEFMH; incluir grau académico e afiliação)

Helena Santa-Clara | PhD | Faculty of Human Kinetics – University of Lisbon.

## Nome do orientador ou responsável científico pelo estudo (se diferente do proponente; incluir grau académico e afiliação)

Helena Santa-Clara | PhD | Faculty of Human Kinetics – University of Lisbon.

## Nome da pessoa de contacto (p. ex., coordenador de projeto, secretariado) (se diferente do proponente)

Vitor Giatte Angarten | PhD student | Faculty of Human Kinetics – University of Lisbon.

## Contactos (email e telefone que o CEFMH utilizará para questões ou correspondência)

Email: vitora@campus.ul.pt

Telefone: +351 914180515

## Nome de outros co-investigadores do estudo (se aplicável) (incluir grau académico e afiliação institucional)

Rita Pinto | MSc | Faculty of Human Kinetics – University of Lisbon.

Vanessa Santos | MSc | Faculty of Human Kinetics – University of Lisbon.

Xavier Melo | PhD | Faculty of Human Kinetics – University of Lisbon.

João L. Marôco | PhD student | University of Massachusetts Boston

Bo Fernhall | PhD | University of Massachusetts Boston

## Indicação da forma de recrutamento e critérios de elegibilidade (Recrutiment methods and Eligibility criteria)

Employing some forms of dissemination (leaflets, flyers, posters, websites, etc.), possible participants will learn about this research project.

Two distinct groups will be enrolled in the proposed study: 1) Patients with CAD, and 2) the age-matched control group. For the CAD group, patients will be recruited mainly from the cardiac rehabilitation program, “Centro de Reabilitação Cardiovascular da Universidade de Lisboa (CRECUL)”, which is located in the “Academia de Fitness” belonging to the “Estádio Universitário”. The aged-matched control group will be recruited from “Estádio Universitário de Lisboa” gyms. From these specific Institutions, Cardiologists and Health Professionals will be contacted directly to identify the inclusion criteria and assist in the recruitment process. Exercise Physiologists from “Estádio Universitário de Lisboa” will be contacted to identify possible “match participants”. After that, the Exercise Physiologists will contact the Researcher who will ask for an appointment to show all the project details. All the inclusion and exclusion criteria for both are described below:

Patients with CAD

- Inclusion diagnosis criteria: Male and Female (only post-menopause females will be included); patients aged 50-80 years old; classified according to the risk stratification criteria for patients with cardiovascular diseases [1]; in the last year predominant sedentary lifestyle; as low and/or moderate risk stratification; having at least one of the following documented stenosis for less than 3 months past≥50% in at least one major coronary artery; prior history of myocardial infarction (MI), percutaneous coronary intervention (PCI), or coronary artery bypass graft (CAGB) surgery

- Exclusion criteria: cardiac surgical procedure or MI or CABG or PCI within >3 months; heart failure diagnosis; documented severe chronic obstructive pulmonary disease; unstable angina; uncontrolled hypertension; uncontrolled atrial arrhythmia or ventricular dysrhythmia; insulin-requiring diabetes mellitus; obese individuals with movement restriction; and any musculoskeletal abnormality that would limit exercise participation; presence or history of neoplasias, neurodegenerative diseases, mood disorders, orthopedic or lung disorders

Aged-matched controls

- Inclusion criteria (age-sex-healthy-trained match): Male and Female (only post-menopause females will be included) who during the last year the individual had at least one medical appointment that did not have more than 2 risk factors for cardiovascular disease and reported any kind of diagnostic or chronic disease; Physically active individual means more than 6 months in a supervised training program performed at least 3 times per week. The training must include combined exercise, resistance and aerobic exercises in the same training session

- Exclusion criteria: currently smokers; diagnosis of cardiovascular disease, presence of orthopaedic diseases with any movement limitation; presence or history of neoplasms; neurodegenerative diseases; mood disorders; lung disorders; medication use (beta blockers, glucocorticoids, antidepressants, etc.); any kind of supplementation, more than two risk factors to CVDs; more than 2 risk factor for cardiovascular diseases; Moreover, if participants showed any kind of negative or abnormal responses during the cardiopulmonary exercise testing test, following the criteria from American Heart Association - exercise standards responses [2], the individual will be excluded.

## Indicação quanto a eventuais populações especiais (menores, grávidas, pessoas idosas, recémnascidos, pessoas com limitações decisórias, estudantes ou funcionários da FMH, outras)

Participants are patients with established CVD.

## No caso de participantes da FMH, indicação se vai recrutar estudantes de turmas onde lecione ou sobre as quais tem responsabilidade; ou se vai recrutar funcionários que dependem de si hierarquicamente)

We are not planning to recruit participants from FMH.

## Condições especiais

None.

# Plano de Investigação (Research plan)

### Study Design

All assessment moments will be done in 1 week in all groups (Both the physical assessments and efforts performed may cause some discomfort and fatigue):

- Day 1 –Cardiopulmonary Exercise Testing (CPET) will be performed at Pulido Valente Hospital. Additionally, pre and post CPET will be evaluated HRV+PWV in 3 distinct moments (baseline + 10 min post + 30 min post);

- Day 2 – Maximal Strength capacity test at Academia de Fitness at the Estádio Universitário de Lisboa where the program CRECUL is located;

Two more days will be used (combined exercise bout) with 72 hours between them:

- Day 3: Pre and Post moderate intensity combined training will be done and central and brachial blood pressure + arterial stiffness in four distinct moments will be assessed (before starting the exercise session: baseline + after finishing the exercise session: 5-, 15- and 30-min post

- Day 4: Pre and Post high intensity combined training will be done and central and brachial blood pressure + arterial stiffness in four distinct moments will be assessed (before starting the exercise session: baseline + after finishing the exercise session: 5-, 15- and 30-min post

Acute Combined Exercise Bouts

Two different bouts, moderate intensity combined training (MiCT) and high intensity combined training (HiCT) will be performed with 72h between them. Both bouts will be composed of a 10-minute progressive warm up, specific methodology of cycle ergometer exercise and multiset resistance exercise (lat pull down; leg press; chest press; leg curl; low row; leg extension), ending with a 10-minute cooldown of passive stretching training. In both exercise sessions, the warm-up and the cool-down will be the same. Warm-up: cycle ergometer (60-70rpm) until 10% of the first ventilatory threshold (VT1) equivalent parameters (heart rate; Borg scale; Load; talk test). Cool down: passive stretching training using upper and lower limb muscles until joint limit for 20-s. The cyclic exercise will be finished with 2-5min of pedaling and load decreasing before stopping. Training impulse (TRIMP) will be calculated for cyclic exercise to prove the difference between bouts [MiCT= (10´ X phase1)+(20´ X phase2)+(2´ X phase1)] [HiCT= (10´ X phase1)+(4sets X 2´ X phase3)+(3sets X 2´ X phase2)+(2´ X phase1)].

*Specific Training bout:*

* MiCT: 20min of continuous pedaling (60-70rpm) in VT1 equivalent parameters **+** resistance training performed 60% of 1 repetition maximum (1RM), 2 sets, 12 repetitions, 45-s resting interval between sets.

* HiCT: it is composed of high-intensity interval training (HIIT) and high-intensity resistance training. HIIT: 5 repetitions of 2 min at VT2 equivalent parameters with 2 min at VT1 equivalent parameters + resistance training performed 80% of 1RM, 2 sets, 12 repetitions, 60-s resting interval between sets.

Designação da tarefa (Task denomination)

1. Cardiorespiratory fitness

A symptom-limited ramp incremental CPET, following the Clinician’s Guide to Cardiopulmonary Exercise Testing in Adults[3], will be performed on a cycle ergometer (Ergostik, Geratherm Respiratory GmbH, Bad Kissingen, Germany) with breath-by-breath gas exchange measurements. First, all the details about the test will be explained. Meanwhile, the participant´s skin will be prepared for a 12-lead electrocardiogram and Polar H7 chest strap. Afterwards, a spirometry test will be performed to determine the load progress per minute (increasing load every 10-s) by “forced expiratory volume 1” (FEV1) and FEV time (FEVt) variables [4]. Ramp protocol´s test: 2 min rest at baseline gas analysis in sitting bike position followed by 2 min unloaded pedaling (~60rpm) (warm-up) and 2 minutes more at rest. Twelve-lead ECG will be recorded continuously. Blood pressure will be recorded at baseline, every two minutes, at peak exercise and each minute during recovery. Borg scale will be asked each minute and blood oxygen saturation levels (SpO2) will be measured. Each patient will be encouraged to exercise until exhaustion: leg fatigue; pedaling <60 rpm; dyspnea unless clinical criteria for test termination occurred. Although, symptoms and signs that compromise the participant's safety, will be sufficient to finish the test, following guidelines and rules [1, 5]. Peak oxygen capacity will be considered the highest attained VO_2_ during the final 30s of exercise and VT will be estimated by the V-slope method. Heart rate recovery (HRR), as a simple marker of parasympathetic activity, will be calculated as the difference between peak HR and HR one minute later. The recovery period will continue until 6 minutes after peak effort. Chronotropic response (CR) to exercise will be evaluated by the % of HR reserve (HRR) used at peak exercise. CR= [peak HR – resting HR/(220 – age – resting HR)x100]. A failure to use 80% of the HRR is defined as chronotropic incompetence. All patients should achieve a respiratory exchange ratio of >1.10, an indicator of peak effort in the CPET. The CPET will provide clinical information for patient screening and exercise intensity prescription. Variables that will be discussed: oxygen consumption peak (VO_2peak_); VT1; VT2; maximum load achieved; time effort; maximum respiratory exchange ratio**; End-tidal CO_2_ partial pressure**, maximum ventilation; load/min; heart rate baseline and recovery 1-2min; blood pressure baseline and recovery 1-2min; maximum Borg scale classification.

2. CENTRAL BLOOD PRESSURE AND arterial stiffness

Central systolic blood pressure (cSBP) will be measured using non-invasive carotid tonometry (Complior, ALAM Medical, Paris, France) while participants are in a supine position. These carotid waveforms will be calibrated from brachial diastolic blood pressure (bDBP) and mean arterial pressure (i.e., 2/3 bDBP + 1/3 bSBP), which are assumed to remain constant throughout the vascular system. To ensure measurement reliability, a single operator will conduct 2 repeated applanation tonometry measurements on the right side of the body, each consisting of 10 waveforms with quality indexes >90%. The average of the two measurements will be considered for data analysis. The added pressure arriving from wave reflection on SBP will be calculated as the difference between bSBP and cSBP. Brachial BP will be measured in the supine position using an Omron automatic sphygmomanometer (HEM-907-E Omron Health Care, Kyoto, Japan) after a 10-min quiet rest in a dim-light room. Hypertension will be defined according to the ESH guidelines (i.e., bSBP ≥ 140 mmHg and/or bDBP ≥ 90 mmHg).

We will also assess central arterial stiffness via carotid-femoral pulse wave velocity (cf PWV), wherein both carotid and femoral waveforms were collected simultaneously using piezoelectric pressure mechanotransducers (Complior, ALAM Medical, Paris, France). Pulse transit times (PTT) are automatically calculated using the intersect tangent algorithm of the foot-to-foot method, enabling the calculation of cfPWV as the ratio of distance to PTT. Travel time distances will be defined as the taped measured distance over body surfaces between the two recording sites of interest, with the cf distance corrected by a factor of 0.8.

3. mUSCLE STRENGHT TESTING

Maximal strength will be assessed by 1 repetition maximum (1RM) test for each of six weight exercises (order: lat pull down, leg press, chest press, leg curl, low row, leg extension) resistance machines available at Academia de Fitness gym. Participants will warm up before testing for 10-min on the treadmill progressively until HR´s -10% VT1 is detected by CPET. After a 1-min rest period, participants who are already familiarized with resistance machines (Life Fitness Corporation, Franklin Park, IL, USA) and techniques (posture, breathing, avoidance of Valsalva maneuver and laterality power) will perform 8 repetitions of a light load (50% predicted 1RM) at the lat pull down and leg press. After that, the patient will be asked to perform a single repetition until the 1RM is reached (4 attempts maximum). For the first attempt, it will be selected a weight based on the previous effort (4 familiarization sessions) which will allow them to perform 3 repetitions (10RM load + 40-50% of this load). The resting period between attempts will be 3 min at least. The resistance can be increased by 2,5 kg when the subject is near his maximum. Strength will be recorded as the maximal number of kilograms lifted in one full range of motion. Similar 1-RM protocols [6–10] to chronic diseases patients and frail elderly have been published.

4. data base management and reports

This task includes the treatment of the variables obtained during the data collection period. The collected data from each equipment/software will be transposed to a unique computer for the research that has confidentiality and privacy characteristics, with a private password of a single Researcher involved, involving a specific storage area in the computer with a new password. To ensure confidentiality, all data will be stored in a database with individual code identification for each participant. In the process of monitoring the database, no longer than 5 years, the information will be disseminated. Throughout the scientific production, writing papers/posters/abstracts / scientific oral presentations, the results found may be published in the form of group analysis. At no time will be released information with individual identification. It is expected to add the data collected to the database within 2 weeks of the end of the respective evaluation moment and store it at the FMH web server allowing detailed progress reports and back-ups when needed.

Each participant will receive an individual report, which will explain all the outcomes, the physiology of the variables, and health cutoff points to compare and explanation about their outcomes. The reports will be sent by email no more than 2 weeks after the last assessment. In case of any doubt, the participant may contact the team researcher by phone or email, or even get an appointment.

5. STATISTICAL ANALYSIS

All statistical analyses will be conducted using R software, version 4.2.1, with a significant level (α) set at 0.05. Data will be reported as mean (SD) unless otherwise stated. We will test the normality and homogeneity of residuals of all models using the Shapiro-Wilk and Levene tests, respectively. We will also inspect QQ plots using the R performance package. Participant characteristics will be compared using Welch’s independent-sample *t*-tests for numerical outcomes and Fisher’s exact tests for categorical outcomes.

To analyse post-exercise changes in blood pressure and central arterial stiffness (i.e., cfPWV), we will use linear mixed models fitted with restricted maximum likelihood. These models use Satterthwaite’s method to approximate degrees of freedom for the F test from the lmerTest package. The fixed effects in the models will be set as time, condition, and group, while each participant was assigned as a random intercept. We will calculate partial omega squares (*ω^2^*) for main effects and interactions (intensity-by-time; group-by-time; group-by-condition and intensity-by-time-by-group) using sjstats package and interpret them based on Cohen’s [11] benchmarks [small (ω*^2^* < 0.05), medium (ω*^2^* < 0.25), and large ω*^2^* > 0.25) effects sizes]. Linear mixed models will be controlled for the medication, V̇O_2_ _peak,_ HR_peak,_ and HR, which will be entered one by one in the model. Post-hoc comparisons will be conducted using the Bonferroni test of the emmeans package when significant main effects and interactions are detected.

*Individual blood pressure responsiveness to acute combined exercise*

Participants will be classified as either showing relevant (responder) or negligible (non-responder) post-exercise hypotensive or hypertensive responses using the ROPE + HDI decision rule to reject or non-reject the null, a Bayesian method [12, 13]. Briefly, this method estimates the percentage of the highest density interval (HDI, similar to the confidence intervals in frequentist statistics) within the range of values around the null – region of practical equivalence (ROPE). This estimated percentage corresponds to different levels of significance, which guides the classification of post-exercise hypotension or hypertensive response, with non-responders defined as > 99 % of HDI and responders as < 1% within the ROPE [12, 13]. We will also consider an undecided category [% of HDI inside ROPE: ≤ 98% to ≥ 2%].

Both the HDI and the ROPE will be computed with the R package bayestestR [14]. The HDI is calculated as an 89% credible interval and derived from each participant’s posterior normal distribution obtained from 1000 simulations based on the individual post-exercise changes in cSBP and bSBP (both 15 and 30-min) compared to pre-exercise values (delta). [12, 13, 15]. Individual normal distributions will be derived using the R’s rnorm function. The standard deviation of each distribution will be defined as the individual TE * √2, where TE is the technical error calculated as the coefficient of variation * baseline mean of cSBP and bSBP) (Swinton et al. 2018). We will estimate the ROPE as 20% of the baseline cSBP and bSBP standard deviation in each group. The ROPE represents the smallest worthwhile difference [12, 16, 17]. Despite the lack of consensus on how to define a true post-exercise BP response, a methodologically sound approach is that BP changes should surpass the technical error of measurement [18].

REFERENCES

1. Pescatello L et al. (2014) ACSM’S Guidelines for Exercise Testing and Prescription. NINTH EDITION.

2. Fletcher GF, Ades PA, Kligfield P, et al (2013) Exercise Standards for Testing and Training. Circulation 128:

3. Balady GJ, Arena R, Sietsema K, et al (2010) Clinician’s Guide to Cardiopulmonary Exercise Testing in Adults: A Scientific Statement From the American Heart Association. Circulation 122:191–225

4. Pretto JJ, Braun GW, Guy PA (2001) Using baseline respiratory function data to optimize cycle exercise test duration. Respirology 6:287–91

5. Fletcher GF, Ades PA, Kligfield P, et al (2013) Exercise Standards for Testing and Training. Circulation 128:

6. Cadore EL, Moneo ABB, Mensat MM, Muñoz AR, Casas-Herrero A, Rodriguez-Mañas L, Izquierdo M (2014) Positive effects of resistance training in frail elderly patients with dementia after long-term physical restraint. Age (Omaha) 36:801–811

7. Rydwik E, Karlsson C, Frändin K, Akner G (2007) Muscle strength testing with one repetition maximum in the arm/shoulder for people aged 75 + - test-retest reliability. Clin Rehabil 21:258–265

8. Maud PJ, Foster C (2006) Physiological assessment of human fitness. Human Kinetics

9. Featherstone JF, Holly RG, Amsterdam EA (1993) Physiologic responses to weight lifting in coronary artery disease. Am J Cardiol 71:287–92

10. Seo D-I, Kim E, Fahs CA, et al (2012) Reliability of the one-repetition maximum test based on muscle group and gender. J Sports Sci Med 11:221–5

11. Cohen J (1988) Statistical Power Analysis for the Behavioral Sciences, 2nd ed. Lawrence Erlbaum Associates, Hillsdale (NJ)

12. Maturana FM, Schellhorn P, Erz G, Burgstahler C, Widmann M, Munz B, Soares RN, Murias JM, Thiel A, Nieß AM (2021) Individual cardiovascular responsiveness to work-matched exercise within the moderate- and severe-intensity domains. Eur J Appl Physiol 121:2039–2059

13. Kruschke JK (2018) Rejecting or Accepting Parameter Values in Bayesian Estimation. Adv Methods Pract Psychol Sci 1:270–280

14. Makowski D, Ben-Shachar MS, Lüdecke D (2019) bayestestR: Describing Effects and their Uncertainty, Existence and Significance within the Bayesian Framework. J Open Source Softw 4:1541

15. Swinton PA, Hemingway BS, Saunders B, Gualano B, Dolan E (2018) A Statistical Framework to Interpret Individual Response to Intervention: Paving the Way for Personalized Nutrition and Exercise Prescription. Front Nutr. https://doi.org/10.3389/fnut.2018.00041

16. Hecksteden A, Pitsch W, Rosenberger F, Meyer T (2018) Repeated testing for the assessment of individual response to exercise training. J Appl Physiol 124:1567–1579

17. Hecksteden A, Kraushaar J, Scharhag-Rosenberger F, Theisen D, Senn S, Meyer T (2015) Individual response to exercise training - A statistical perspective. J Appl Physiol 118:1450–1459

18. De Brito LC, Fecchio RY, Peçanha T, Lima A, Halliwill J, Forjaz CLDM (2019) Recommendations in Post-exercise Hypotension: Concerns, Best Practices and Interpretation. Int J Sports Med 40:487–497
